# Assisted reproduction in the public sector: why secondary-level hospitals matter for equity and continuity of care

**DOI:** 10.3389/frph.2026.1974930

**Published:** 2026-10-19

**Authors:** Jesús Endara-Mina, Javier Bermúdez-Gavilanes, Franklin-Rafael Salazar, Lisset A. Manzano-Gallegos, Jessica M. Figueroa-Andrade, Dayan N. Ortega-Parraga, David E. González-Naranjo, Fernando Maldonado-Montalvan, Claudia Reytor-González, Daniel Simancas-Racines

**Affiliations:** 1 Facultad de Ciencias Políticas y Jurídicas, Universidad Técnica Particular de Loja, Loja, Ecuador; 2 Teaching and Research Coordination, Hospital Provincial General Pablo Arturo Suárez, Quito, Ecuador; 3 Facultad de Ciencias de la Salud y Bienestar Humano, Universidad Tecnológica Indoamérica, Ambato, Ecuador; 4 Faculty of Health and Social Services, Universidad Estatal de Milagro (UNEMI), Milagro, Ecuador; 5 Facultad de Ciencias de la Salud, Universidad Técnica Estatal de Quevedo (UTEQ), Quevedo, Ecuador; 6 Facultad de Ciencias Sociales y de la Salud, Universidad Estatal Península de Santa Elena, Santa Elena, Ecuador; 7 Facultad de Salud y Bienestar, Pontificia Universidad Católica del Ecuador (PUCE), Quito, Ecuador; 8 Facultad de Ciencias de la Salud, Universidad Iberoamericana del Ecuador (UNIB.E), Quito, Ecuador; 9 Facultad de Ciencias de la Salud, Universidad Nacional Mayor de San Marcos (UNMSM), Lima, Perú; 10 Facultad de Medicina, Universidad de las Américas, Quito, Ecuador

**Keywords:** assisted reproductive technology, equity in healthcare, health systems, infertility, public health policy, public sector, reproductive health, secondary-level hospitals

## Abstract

**Background:**

Infertility is a major public health challenge, yet access to assisted reproductive technology (ART) remains unequal, particularly where public provision is limited. This Policy and Practice Review examines the role of secondary-level hospitals in integrating ART into public health systems.

**Methods:**

A structured literature search was conducted in PubMed/MEDLINE, Scopus, Web of Science, and LILACS, without publication-date restrictions, including literature in English, Spanish, and Portuguese. Evidence on access, service organization, referral pathways, continuity of care, workforce, health information systems, governance, and financing was synthesized thematically and interpreted through expert-informed policy analysis.

**Results:**

The review identified geographic, financial, organizational, and health-system barriers to equitable ART access. Integrated models may allow specialized centers to retain high-complexity procedures while secondary-level hospitals support infertility assessment, referral coordination, follow-up, and pregnancy surveillance. Eight policy priorities were identified: integration of secondary-level hospitals; standardized referral pathways; workforce development; national ART registries; equity-oriented evaluation; patient and community participation; integrated governance and quality assurance; and sustainable financing. An implementation framework specifying responsible actors, minimum resources, implementation phases, barriers, and monitoring indicators was developed.

**Conclusion:**

Integrating secondary-level hospitals into coordinated ART networks may strengthen continuity of care, geographic accessibility, and use of specialized resources. These potential benefits require prospective evaluation during implementation.

## 1 Introduction

Infertility is a public health problem that affects millions of people worldwide and is associated with substantial social, economic, and psychological consequences. Over recent decades, assisted reproductive technology (ART) has transformed the therapeutic options available to people facing infertility. However, access to these services remains limited and unequal, particularly in low- and middle-income countries, where high costs restrict availability for large segments of the population (1).

In Ecuador, the legislative debate on a regulatory framework for ART represents an important opportunity to advance sexual and reproductive rights (2). However, the discussion has focused mainly on the economic, bioethical, legal, and regulatory aspects of high-complexity procedures, while an equally decisive question has received less attention: how to organize the public provision of these services and ensure effective, equitable, and sustainable access (3).

Implementing public ART services is not limited to enabling specialized centers capable of performing *in vitro* fertilization (IVF) or intracytoplasmic sperm injection (ICSI). It also requires financing mechanisms, eligibility criteria, referral pathways, and clear responsibilities across levels of care. The success of these policies will depend on the capacity of the health authority to coordinate the health care network, ensure continuity of care, and reduce the geographic, economic, and social barriers that have historically shaped access to specialized services (4).

This Policy and Practice Review examines policy options for organizing assisted ART within public health systems, with particular emphasis on the role of secondary-level hospitals in integrated care networks. We analyze how different service delivery models may influence equity, continuity of care, resource allocation, and health system performance, and propose actionable recommendations to support the implementation of publicly funded ART programs. Specifically, the review addresses three main questions: (1) How can ART services be organized across different levels of public health systems while preserving the concentration of high-complexity procedures in specialized centers? (2) What potential role can secondary-level hospitals play in improving access, referral coordination, continuity of care, and health-system integration across the infertility care pathway? and (3) What policy, governance, financing, workforce, information-system, and implementation mechanisms are required to operationalize an integrated network-based model for publicly funded ART?

## 2 Methods

### 2.1 Study design

This study was conducted as a Policy and Practice Review. The methodological approach combined a structured review of relevant literature with an expert-informed policy analysis. The purpose was to synthesize evidence relevant to the organization of publicly funded ART services and to translate this evidence into actionable health-system and policy recommendations, with particular emphasis on the potential role of secondary-level hospitals within integrated care networks. The review was not designed as a systematic review or meta-analysis, and no review protocol was registered.

### 2.2 Operational definition of secondary-level hospitals

For the purposes of this review, secondary-level hospitals were operationally defined as hospital facilities providing specialist outpatient and inpatient care beyond the primary-care level and serving as referral points within an organized healthcare network. Consistent with World Health Organization classifications (5), these facilities are generally located at the district or regional level, provide multiple clinical specialties, and may be designated across health systems as district, provincial, regional, or general hospitals. In the Ecuadorian health system (6), the hospital-based component of the second level of care comprises basic and general hospitals. Accordingly, this review uses the term “secondary-level hospital” as a functional rather than exclusively administrative designation, referring to hospitals capable of providing specialist assessment, diagnostic and therapeutic support, hospitalization, and referral coordination, while excluding tertiary or highly specialized centers responsible for high-complexity procedures.

### 2.3 Literature search and evidence selection

A structured literature search was conducted in PubMed/MEDLINE, Scopus, Web of Science, and LILACS, with the final search conducted on 8 June 2026. No publication-date restrictions were applied. The search included literature published in English, Spanish, and Portuguese to capture evidence from diverse health-system contexts, including experiences from Latin America that may be underrepresented in English-language literature.

The search focused on literature addressing access and equity, organization of services across levels of care, referral and counter-referral pathways, continuity and coordination of care, workforce capacity, health information systems and ART registries, governance, financing, and implementation of integrated healthcare networks. Search concepts were developed using combinations of controlled vocabulary, including Medical Subject Headings (MeSH) where applicable, and free-text terms. Core concepts included “Infertility,” “Reproductive Techniques, Assisted,” “Delivery of Health Care, Integrated,” “Continuity of Patient Care,” “Referral and Consultation,” “Health Services Accessibility,” “Health Equity,” “Hospitals,” “Health Policy,” “Health Planning,” “Health Personnel,” “Health Information Systems,” “Registries,” “Health Care Costs,” and “Universal Health Coverage.” These were complemented by free-text terms including *assisted reproductive technology*, *ART*, *fertility services*, *infertility care*, *secondary-level hospitals*, *secondary care*, *tertiary care*, *referral*, *counter-referral*, *integrated care*, *public health system*, *publicly funded*, *governance*, *financing*, *equity*, and *access*. Search terms and controlled vocabulary were adapted to the indexing structure and syntax of each database, including equivalent terminology in Spanish and Portuguese where appropriate.

Peer-reviewed original studies, reviews, policy analyses, relevant institutional reports, and guidance documents were considered when they directly informed the policy questions addressed by the review. Evidence was selected according to its relevance to the organization, accessibility, coordination, financing, governance, and implementation of ART services rather than for quantitative synthesis. Particular attention was given to evidence describing health-system organization, barriers to ART access, integrated service delivery, referral coordination, continuity across the reproductive care pathway, and policy or implementation considerations relevant to publicly funded services.

### 2.4 Evidence synthesis and policy analysis

The selected evidence was synthesized thematically according to the principal health-system domains identified in the review, including access and equity, service organization, referral and counter-referral, continuity of care, workforce capacity, health information systems, governance, financing, and implementation. Rather than estimating pooled effects, the synthesis aimed to identify recurring organizational barriers, policy gaps, relevant service-delivery approaches, and potential strategies for integrating ART into public health systems.

The evidence identified through the literature review was subsequently analyzed within the Public Policy Observatory of Provincial General Pablo Arturo Suárez Hospital, with contributions from public health experts affiliated with several universities in Ecuador. Expert input supported the contextual interpretation of the evidence from health-system and public policy perspectives and its translation into actionable recommendations. This process was intended to complement the evidence identified in the literature and did not constitute a formal expert-consensus method.

### 2.5 Development of policy recommendations

Policy recommendations were developed by integrating the findings of the literature review with the expert-informed policy analysis. Recommendations were formulated around the main organizational and implementation challenges identified across the evidence and were structured to address the distribution of functions across levels of care, referral and counter-referral mechanisms, workforce development, health information systems, equity, stakeholder participation, governance, and sustainable financing.

To facilitate their translation into practice, the recommendations were organized into an implementation framework specifying the policy rationale, responsible actors and implementation mechanisms, minimum resource requirements, implementation phases, potential barriers, and suggested monitoring indicators. The proposed framework was designed to support adaptation to different health-system contexts rather than prescribe a single organizational model.

## 3 Assessment of policy options

The expansion of ART within publicly funded health systems requires policy decisions that extend beyond the regulation of clinical procedures or financing mechanisms (7). Governments must also determine how ART services should be organized across different levels of care to maximize equity, efficiency, and continuity of care. Although the implementation of ART has traditionally focused on establishing highly specialized tertiary fertility centers, increasing attention has been directed toward health system models capable of integrating specialized reproductive services within broader healthcare networks (8).

Highly centralized models concentrate IVF, ICSI, and other advanced reproductive procedures in tertiary hospitals or dedicated fertility centers. This strategy is justified by the substantial infrastructure, laboratory requirements, specialized equipment, and multidisciplinary expertise needed to perform complex ART procedures safely and efficiently (9). Centralization may also facilitate quality assurance, clinical standardization, and optimization of expensive technological resources.

However, exclusive reliance on centralized services may inadvertently reinforce existing inequalities in access to infertility care. In countries characterized by marked geographic, economic, and social disparities, patients frequently face long travel distances, repeated referrals, transportation expenses, accommodation costs, and loss of income during treatment (10, 11). These indirect barriers may delay treatment initiation, reduce adherence to clinical recommendations, and disproportionately affect individuals living in rural or underserved areas. Consequently, the organization of ART services should not be evaluated solely according to technical efficiency but also according to its capacity to promote equitable access across the entire population (12).

Infertility management is inherently longitudinal and extends well beyond the performance of ART procedures. The continuum of care includes early clinical evaluation, diagnostic assessment, identification and treatment of underlying reproductive disorders, counseling, referral to specialized fertility centers, monitoring during treatment, prenatal care following successful conception, and surveillance of maternal and neonatal outcomes (13). Fragmentation across these stages may result in delayed diagnosis, duplication of diagnostic tests, inefficient referrals, discontinuity of care, and loss to follow-up, ultimately compromising both clinical outcomes and patient experience (14).

Within this context, integrated network-based models have emerged as a promising organizational alternative for publicly funded ART programs. Rather than concentrating every component of infertility care in specialized centers, these models distribute responsibilities across different levels of the health system according to their respective capabilities. Specialized fertility centers continue to provide high-complexity reproductive procedures, whereas secondary-level hospitals assume complementary responsibilities related to infertility assessment, coordination of referrals, longitudinal follow-up, and integration with maternal and neonatal healthcare services.

This network-based model distributes responsibilities across levels of care while maintaining continuity throughout the reproductive pathway (Figure 1).

**Figure 1:**
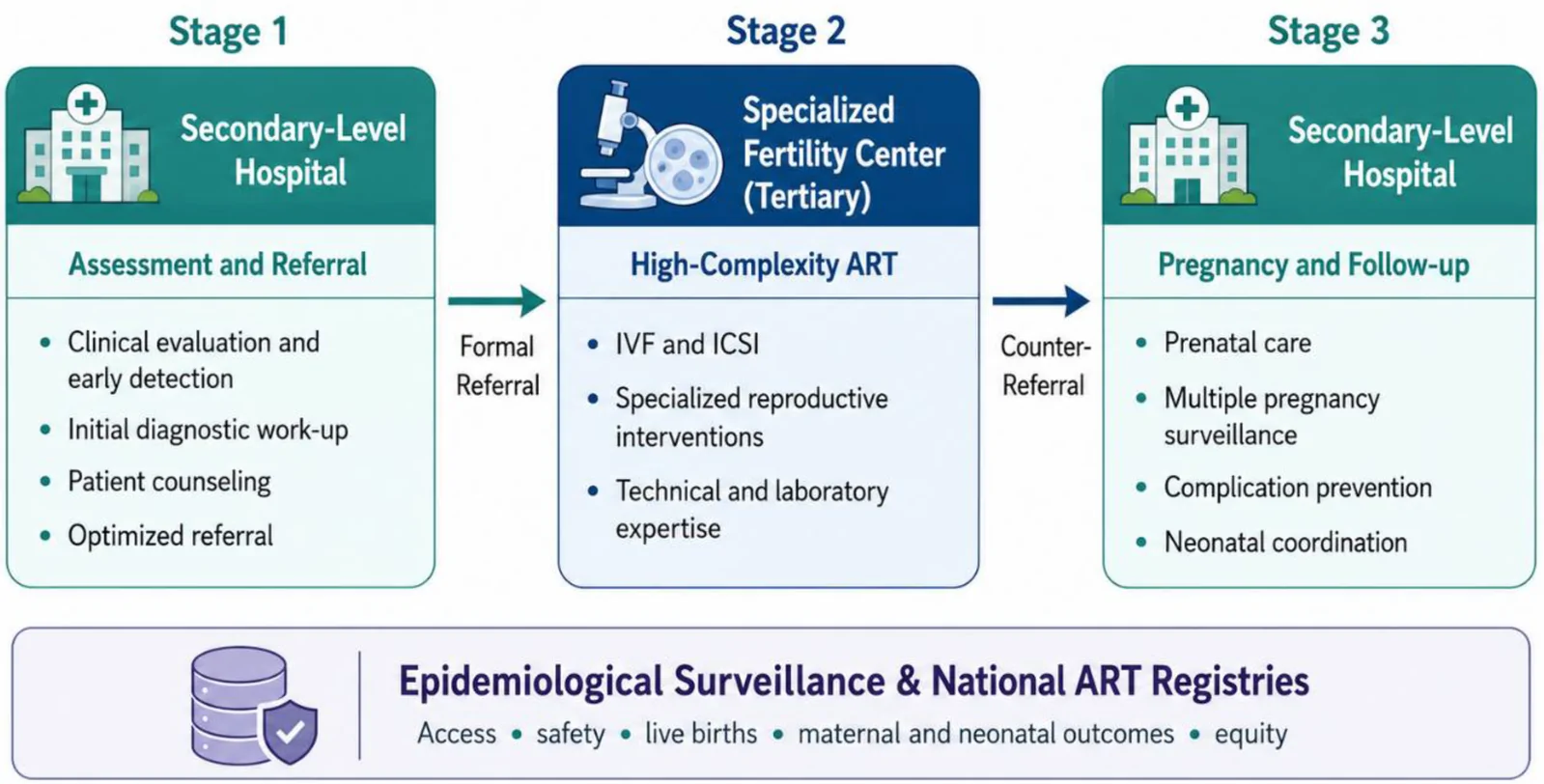
Integrated network-based model for continuity of care in assisted reproduction. ART, assisted reproductive technology; IVF, *in vitro* fertilization; ICSI, intracytoplasmic sperm injection.

Secondary-level hospitals are particularly well positioned to fulfill these functions because, in many health systems, they constitute the principal specialized referral facilities for large segments of the population and possess considerably broader territorial coverage than tertiary hospitals (15, 16). Their participation may begin during the earliest stages of infertility management through comprehensive clinical evaluation, initial diagnostic work-up, identification of reproductive risk factors, management of common gynecological disorders, and patient counseling. By resolving less complex clinical problems locally and referring only appropriately selected patients for advanced ART procedures, secondary-level hospitals may optimize referral pathways and improve the allocation of highly specialized resources (17).

Their contribution extends beyond referral. Following ART treatment, secondary-level hospitals may facilitate continuity of care by coordinating communication with fertility centers, monitoring pregnancies achieved through ART, providing routine prenatal care, identifying pregnancy-related complications, supervising multiple gestations, and coordinating neonatal services when necessary (18). Beyond clinical coordination, integrated care networks may also strengthen health system governance (19). Establishing formal referral and counter-referral pathways between secondary-level hospitals and specialized fertility centers facilitates standardized clinical protocols, improves communication between providers, and generates more complete information regarding patient trajectories throughout infertility treatment (19, 20).

Another important advantage of integrated models is their potential contribution to health information systems. Coordination between secondary-level hospitals and tertiary fertility centers could support the development of national ART registries capable of monitoring pregnancy rates, live birth rates, obstetric complications, prematurity, neonatal outcomes, and indicators of equitable access. Such information is fundamental for evaluating program effectiveness, identifying regional disparities, guiding resource allocation, and informing future policy decisions (21–23).

Although network-based models appear particularly attractive for countries seeking to expand publicly funded ART, successful implementation requires careful consideration of several operational dimensions. These include governance arrangements, financing mechanisms, standardized eligibility criteria, workforce capacity, referral protocols, interoperability of health information systems, and quality assurance across different levels of care (24, 25). The effectiveness of these organizational strategies ultimately depends on the ability of health authorities to coordinate integrated healthcare networks while maintaining high standards of clinical quality and equitable access (19).

## 4 Policy implications

### 4.1 Equity in access to assisted reproductive technology

Ensuring equitable access to ART remains one of the principal challenges for publicly funded infertility services. Although concentrating highly specialized procedures in tertiary fertility centers may optimize technical expertise and infrastructure utilization, such models may also widen existing geographic and socioeconomic disparities (26). Individuals living in rural or remote areas often experience greater travel distances, increased transportation and accommodation costs, and substantial indirect expenses associated with repeated clinical visits. These barriers may delay treatment initiation, reduce adherence to care, and disproportionately affect populations already facing structural inequities in access to specialized healthcare (26, 27).

An integrated service delivery model that incorporates secondary-level hospitals may partially mitigate these disparities by decentralizing the components of infertility care that do not require advanced reproductive laboratories. Initial evaluation, diagnostic testing, counseling, follow-up consultations, and pregnancy monitoring can be delivered closer to patients' communities while reserving highly complex procedures for specialized fertility centers. Such an approach may reduce financial barriers, improve accessibility, and promote more equitable utilization of publicly funded ART services (24).

However, the contribution of secondary-level hospitals should not be interpreted as sufficient to address all barriers to ART access. Their integration may primarily reduce geographic and organizational barriers by bringing initial assessment, diagnostic testing, counseling, follow-up, and pregnancy monitoring closer to patients' communities, while also reducing some indirect costs associated with repeated travel to tertiary centers (28–30). In contrast, broader financial barriers related to the affordability of ART procedures, socioeconomic inequalities, shortages of specialized personnel, restrictive eligibility criteria, and limitations in reproductive laboratory capacity cannot be resolved through service decentralization alone (31). Addressing these barriers requires complementary measures, including sustainable public financing, equitable eligibility policies, workforce development, expansion of specialized capacity, and coordinated national governance (1).

Beyond geographic accessibility, equity should also be understood in terms of timely access, continuity of care, and the ability of health systems to provide coordinated services throughout the infertility care pathway. Public ART programs should therefore incorporate organizational strategies that minimize unnecessary referrals, reduce waiting times, and facilitate navigation across different levels of care (32).

### 4.2 Health system efficiency and resource allocation

Public financing of ART requires careful allocation of limited healthcare resources. Establishing advanced reproductive laboratories in every hospital is unlikely to be economically feasible in most low- and middle-income countries. Consequently, health systems must identify organizational models that maximize efficiency without compromising equitable access (33).

Integrated care networks offer the possibility of distributing responsibilities according to institutional capacity. Specialized fertility centers can concentrate high-complexity interventions, whereas secondary-level hospitals can perform clinical assessment, infertility investigations, management of common reproductive disorders, and long-term follow-up. This functional distribution may optimize utilization of highly specialized services by reducing unnecessary referrals and allowing tertiary centers to focus on patients requiring advanced reproductive interventions (24, 33, 34).

Furthermore, coordinated referral systems may improve patient flow across the healthcare network, facilitate more efficient scheduling of ART procedures, and reduce duplication of diagnostic tests. These organizational efficiencies may ultimately contribute to more sustainable public financing of infertility services while preserving quality of care (33, 34).

### 4.3 Continuity of care across the reproductive pathway

Infertility should not be regarded as an isolated clinical episode but rather as a continuum of care that frequently extends over several months or years. Patients require multiple interactions with healthcare providers before, during, and after ART treatment, including diagnostic evaluation, counseling, treatment of associated medical conditions, specialized referral, prenatal care, and neonatal follow-up (22, 35). Fragmented service delivery may compromise both patient experience and clinical outcomes. Poor communication between providers, lack of standardized referral pathways, and insufficient follow-up mechanisms may result in delays, repeated investigations, discontinuity of treatment, and loss to follow-up (36).

Integrating secondary-level hospitals into ART networks may strengthen continuity of care by providing a permanent clinical interface between patients and specialized fertility centers. These hospitals can facilitate coordination throughout the reproductive pathway while maintaining long-term relationships with patients before conception, during pregnancy, and after delivery. Such continuity may also improve management of pregnancy-related complications and promote better maternal and neonatal outcomes (37, 38).

### 4.4 Governance, quality assurance, and health information systems

The expansion of publicly funded ART also requires robust governance structures capable of coordinating services across multiple institutions. Clearly defined referral pathways, standardized clinical protocols, and effective communication mechanisms are essential to ensure consistency in clinical practice and equitable access to care (24).

Integrated networks also create opportunities to strengthen health information systems. National ART registries could collect standardized information on treatment utilization, pregnancy rates, live births, maternal complications, neonatal outcomes, and indicators of equity in access. These data are fundamental for evaluating program performance, identifying regional disparities, monitoring safety, and supporting evidence-informed policy decisions (22, 39).

Moreover, continuous monitoring of clinical indicators may facilitate quality improvement initiatives and enhance accountability within publicly funded ART programs. Transparent reporting systems would allow policymakers to evaluate whether public investment in infertility care translates into improved health outcomes and reduced inequalities (40).

### 4.5 Implications for future public policy

As more countries consider incorporating ART into universal health coverage, policy discussions should move beyond the regulation of reproductive technologies themselves and address how infertility services are organized within national health systems. Organizational design is likely to influence not only clinical effectiveness but also financial sustainability, territorial equity, patient experience, and long-term health system performance (41).

Future public policies should therefore adopt a systems perspective that recognizes infertility care as a coordinated continuum rather than a single specialized intervention. Integrating secondary-level hospitals within network-based models represents one potential strategy for strengthening accessibility, improving continuity of care, and optimizing the use of specialized reproductive services. Nevertheless, successful implementation will require context-specific adaptations according to available infrastructure, workforce capacity, financing mechanisms, and existing referral systems (7, 19).

## 5 Actionable recommendations

The successful integration of ART into publicly funded health systems requires organizational strategies that extend beyond the provision of high-complexity reproductive procedures. Drawing on the evidence and policy considerations reviewed above, the following recommendations identify potential strategies to strengthen equitable access, continuity of care, coordination across levels of care, and health system performance. Their implementation should be adapted to national and local contexts, considering existing health system structures, available resources, and service-delivery capacity.

### 5.1 Recommendation 1. Integrate secondary-level hospitals into national ART service delivery networks

Public ART programs should formally recognize secondary-level hospitals as essential components of integrated reproductive healthcare networks. These facilities should assume responsibility for infertility assessment, initial diagnostic evaluation, patient counseling, referral coordination, follow-up after ART treatment, and pregnancy surveillance, while highly specialized fertility centers continue to provide advanced reproductive procedures such as IVF and ICSI (19, 24). Clearly defined responsibilities across different levels of care are expected to improve coordination and may contribute to more efficient resource allocation and fewer unnecessary referrals; however, these effects require prospective evaluation within publicly funded ART programs.

### 5.2 Recommendation 2. Develop standardized referral and counter-referral pathways

National ART programs should establish standardized referral protocols that define patient eligibility, referral criteria, communication mechanisms between institutions, and responsibilities throughout the continuum of infertility care (42, 43). These pathways should explicitly assign responsibility for patient navigation and psychosocial support. Designated care coordinators or trained healthcare professionals at secondary-level hospitals could serve as the primary point of contact for patients, supporting appointment coordination, referral and counter-referral processes, access to information, and continuity across levels of care (44). Psychosocial support should be provided by appropriately trained professionals, including psychologists or other qualified members of multidisciplinary teams, with referral to specialized mental health services when required (45).

### 5.3 Recommendation 3. Strengthen workforce capacity at the secondary level

The expansion of public ART services should be accompanied by targeted training programs for healthcare professionals working in secondary-level hospitals. Capacity-building initiatives should include infertility assessment, reproductive endocrinology, fertility preservation counseling, management of common gynecological conditions associated with infertility, and coordination of multidisciplinary care (19, 46).

### 5.4 Recommendation 4. Establish national ART registries and monitoring systems

Health authorities should develop national registries capable of collecting standardized information on ART utilization, pregnancy outcomes, live birth rates, maternal complications, neonatal outcomes, and indicators of equitable access. Robust health information systems are essential for evaluating program effectiveness, monitoring safety, identifying regional disparities, and supporting evidence-informed decision-making (22).

### 5.5 Recommendation 5. Incorporate equity indicators into policy evaluation

The success of public ART policies should not be measured exclusively through clinical outcomes such as pregnancy or live birth rates. Evaluation frameworks should also include indicators related to equity, including geographic accessibility, waiting times, socioeconomic disparities in service utilization, continuity of care, treatment completion, and patient-reported experience measures (47).

### 5.6 Recommendation 6. Incorporate patient and community participation into ART program design and evaluation

Public ART programs should establish formal mechanisms for patient and community participation throughout program design, implementation, and evaluation. Patient representatives, community organizations, and advocacy groups could participate in advisory committees, periodic consultations, and the development and review of patient-reported experience measures, referral pathways, and accessibility strategies. Importantly, engagement mechanisms should extend beyond current ART users to deliberately include individuals who were unable to access treatment, were deemed ineligible, experienced substantial delays, or discontinued treatment. Their perspectives may identify financial, geographic, organizational, cultural, and informational barriers that are not adequately captured by clinical outcomes or data from patients who successfully navigate ART services. Including these perspectives may therefore support more responsive and equitable service delivery and inform targeted strategies to reduce treatment non-initiation and discontinuation (48, 49).

### 5.7 Recommendation 7. Promote integrated governance for infertility care

Effective ART policies require coordination among ministries of health, specialized fertility centers, secondary-level hospitals, professional societies, and regulatory agencies. Governance frameworks should clearly define institutional responsibilities, financing mechanisms, accreditation standards, quality assurance processes, and data-sharing agreements (33). A coordinated governance model may facilitate implementation, strengthen accountability, and ensure that public investment in ART translates into sustainable improvements in reproductive healthcare delivery (25).

To translate these policy recommendations into operational strategies, Table 1 presents an implementation framework summarizing the proposed actions, their policy rationale, responsible actors and implementation mechanisms, minimum resource requirements, potential barriers, and suggested indicators for monitoring progress and performance. The framework also adopts a phased approach to implementation. Phase 1 focuses on planning, capacity assessment, and the definition of institutional responsibilities; Phase 2 involves initial implementation within selected services or healthcare networks; and Phase 3 encompasses progressive scale-up, routine monitoring, evaluation, and continuous quality improvement. The timing and scope of each phase should be adapted to the available infrastructure, workforce capacity, financing mechanisms, and organizational characteristics of each health system.

**Table 1:** Policy recommendations and implementation framework for integrating assisted reproductive technology into public health systems.

Recommendation	Policy rationale	Responsible actors and implementation mechanisms (Who? What? How?)	Minimum resources	Implementation phases	Potential barriers	Suggested indicators
Integrate secondary-level hospitals into ART care networks	Network-based models may improve continuity of care, optimize referrals, and reduce geographic inequities in access to infertility services (8–11).	**Who:** Ministry of Health, regional health authorities, tertiary fertility centers, secondary hospitals. **What:** Define roles for infertility assessment, referral, follow-up, pregnancy surveillance, and care coordination. **How:** National service delivery protocols and integrated care pathways.	Designated ART referral focal point; trained clinical personnel; basic diagnostic capacity for infertility assessment; standardized clinical and referral protocols; communication mechanisms with tertiary fertility centers; and a basic system for referral and follow-up tracking.	**Phase 1:** Map existing secondary and tertiary capacity, identify gaps, and define institutional roles. **Phase 2:** Implement standardized pathways in selected hospitals and establish coordination with fertility centers. **Phase 3:** Progressively expand the network and routinely evaluate access, referrals, continuity, and outcomes.	Limited institutional coordination; variability in infrastructure; resistance to organizational change.	Number and proportion of eligible secondary-level hospitals integrated into ART networks; referral completion rate; median patient travel distance to ART-related services; median waiting time from initial infertility assessment to referral; proportion of patients receiving follow-up at secondary-level hospitals.
Develop standardized referral and counter-referral pathways	Standardized referral systems improve coordination, reduce unnecessary referrals, and facilitate continuity of care (7, 10).	**Who:** Ministry of Health, fertility specialists, obstetric and gynecological societies. **What:** National referral criteria, communication protocols, clearly assigned responsibility for patient navigation, and access to psychosocial support throughout the care pathway. **How:** Clinical guidelines integrated into routine practice, with designated care coordinators or trained healthcare professionals at secondary-level hospitals serving as points of contact and multidisciplinary teams providing or coordinating psychosocial support.	Standardized referral criteria and protocols; designated care coordinators or trained healthcare professionals; communication channels between facilities; referral-tracking mechanisms; and access to psychosocial support.	**Phase 1:** Develop referral criteria, responsibilities, patient-navigation procedures, and communication protocols. **Phase 2:** Pilot referral and counter-referral pathways and train responsible personnel. **Phase 3:** Scale up standardized pathways and monitor referral appropriateness, completion, waiting times, and continuity of care.	Lack of interoperability between institutions; heterogeneous clinical practices.	Referral appropriateness rate; referral completion rate; counter-referral completion rate; median time from infertility diagnosis to first fertility-center consultation; proportion of referred patients with documented care coordination; proportion of patients lost to follow-up during the referral pathway.
Strengthen workforce capacity at secondary-level hospitals	Adequately trained professionals improve infertility diagnosis, counseling, referral quality, and follow-up.	**Who:** Universities, professional societies, Ministries of Health. **What:** Continuing education in infertility evaluation, reproductive endocrinology, counseling, and coordinated care. **How:** Certification programs, clinical training, and periodic competency assessments.	Training materials; qualified trainers; protected training time; competency-assessment tools; and access to continuing education in infertility assessment, counseling, referral, and coordinated care.	**Phase 1:** Assess workforce competencies and identify training needs. **Phase 2:** Implement targeted training and competency assessment. **Phase 3:** Establish periodic continuing education, reassessment, and quality-improvement activities.	Workforce shortages; unequal access to continuing education; financial constraints.	Number and proportion of relevant healthcare professionals completing ART-related training; proportion achieving predefined competency standards; frequency of competency reassessment; adherence to standardized infertility assessment and referral protocols.
Establish a national ART registry	Comprehensive health information systems support monitoring of clinical outcomes, quality of care, and equitable access.	**Who:** Ministry of Health, fertility centers, secondary hospitals, national statistics agencies. **What:** National registry including ART cycles, pregnancy outcomes, maternal and neonatal complications, and equity indicators. **How:** Standardized electronic reporting systems with mandatory participation.	Standardized minimum dataset; electronic reporting platform; trained data personnel; data governance and protection procedures; and mechanisms for reporting from participating facilities.	**Phase 1:** Define the minimum dataset, governance standards, reporting responsibilities, and technical requirements. **Phase 2:** Pilot standardized reporting in selected fertility centers and secondary hospitals. **Phase 3:** Expand nationally and routinely assess data completeness, quality, and use for policy evaluation.	Incomplete reporting; fragmented health information systems; data protection challenges.	Proportion of ART-providing facilities reporting to the national registry; completeness and timeliness of mandatory reporting; number of ART cycles recorded; clinical pregnancy rate per initiated ART cycle; live birth rate per initiated ART cycle; rates of multiple pregnancy, maternal complications, prematurity, and adverse neonatal outcomes.
Incorporate equity indicators into program evaluation	Program performance should be evaluated beyond pregnancy rates to assess equitable access and continuity of care.	**Who:** Ministry of Health, public health observatories, researchers. **What:** Develop equity indicators according to geographic location, socioeconomic status, ethnicity, waiting times, and treatment completion. **How:** Routine analysis integrated into national monitoring frameworks.	Standardized equity indicators; capacity to collect disaggregated data; analytical personnel; routine monitoring tools; and mechanisms for reporting geographic, socioeconomic, ethnic, access, and treatment-completion measures.	**Phase 1:** Define and standardize equity indicators and identify available data sources. **Phase 2:** Integrate indicators into routine monitoring and establish baseline measurements. **Phase 3:** Analyze trends and disparities periodically and use findings to guide corrective policy actions.	Limited availability of disaggregated data; inconsistent indicator definitions.	Median waiting time from referral to first fertility consultation; referral completion rate; median out-of-pocket patient costs associated with ART-related care, including travel and accommodation; treatment initiation and completion rates; geographic coverage of ART services; and absolute and relative differences in access, waiting times, referral completion, patient costs, and ART utilization across geographic, socioeconomic, and ethnic groups.
Strengthen governance and quality assurance	Effective ART programs require coordinated governance, accreditation, and continuous quality improvement.	**Who:** Ministry of Health, regulatory agencies, scientific societies. **What:** Accreditation standards, quality indicators, multidisciplinary coordination, periodic audits. **How:** National regulatory framework with continuous monitoring and external evaluation.	Defined governance structure; accreditation and quality standards; designated coordinating authority; standardized quality indicators; audit mechanisms; and institutional communication channels.	**Phase 1:** Define governance responsibilities, accreditation standards, and quality indicators. **Phase 2:** Implement coordination mechanisms, accreditation processes, and periodic audits. **Phase 3:** Maintain continuous monitoring, external evaluation, accountability, and quality-improvement cycles.	Regulatory fragmentation; insufficient financial resources; variability in quality standards.	Proportion of ART programs with formal patient and community participation mechanisms; proportion of participating representatives from underserved groups; participation of individuals who did not initiate or complete treatment; documented reasons for treatment non-initiation and discontinuation; proportion of patient- and community-informed recommendations incorporated into program improvement; patient-reported experience measures.
Incorporate patient, community, and frontline healthcare staff perspectives into ART program design and evaluation	Patient, community, and frontline healthcare staff perspectives may identify barriers to access, treatment initiation, continuity of care, feasibility, care coordination, and treatment completion that are not adequately captured by routine clinical or administrative data. Including individuals who could not access or complete treatment, as well as healthcare professionals involved in service delivery, may strengthen the equity, feasibility, responsiveness, and person-centeredness of ART programs.	**Who:** Ministry of Health, patient representatives, community organizations, advocacy groups, frontline healthcare professionals, and healthcare providers. **What:** Incorporate patient, community, and frontline healthcare staff perspectives into ART program design, implementation, and evaluation, specifically including individuals who were unable to access treatment, were deemed ineligible, experienced substantial delays, or discontinued treatment, as well as professionals involved in infertility assessment, referral, treatment, and follow-up. **How:** Patient advisory committees, multidisciplinary staff advisory groups, periodic community and staff consultations, patient-reported experience measures, and targeted surveys or interviews with patients and healthcare professionals.	Patient/community and healthcare staff engagement mechanisms; designated participation focal point; accessible consultation tools; patient-reported experience measures; staff feedback mechanisms; and mechanisms to identify and engage individuals who could not access or complete treatment.	**Phase 1:** Identify relevant patient, community, and frontline healthcare staff representatives, including underserved and non-completing populations, and establish participation mechanisms. **Phase 2:** Implement advisory committees, multidisciplinary staff consultations, surveys, or interviews and incorporate stakeholder feedback into program design and implementation. **Phase 3:** Institutionalize stakeholder participation and periodically evaluate whether patient, community, and healthcare staff input results in measurable service improvements.	Limited representation of underserved populations; difficulties identifying and engaging individuals who did not access or discontinued treatment; limited staff time and participation; participation burden; and limited institutional mechanisms for incorporating stakeholder feedback into policy and organizational decisions.	Proportion of ART programs with formal patient, commun`ity, and healthcare staff participation mechanisms; representation of underserved groups; participation of individuals who did not initiate or complete treatment; proportion of relevant frontline healthcare staff participating in consultation activities; documented patient- and staff-identified barriers; proportion of stakeholder-informed recommendations incorporated into program improvement; and patient- and staff-reported experience measures.
Promote sustainable financing mechanisms	Long-term sustainability requires financing strategies that balance equity, efficiency, and fiscal responsibility.	**Who:** Ministry of Health, Ministry of Finance, national insurance agencies. **What:** Define eligibility criteria, reimbursement models, and long-term funding mechanisms. **How:** Integration within universal health coverage and periodic economic evaluation.	Dedicated planning and health-economics capacity; expenditure and utilization data; defined eligibility criteria; reimbursement framework; and mechanisms for budget allocation and financial monitoring.	**Phase 1:** Estimate resource requirements, assess financing options, and define eligibility and coverage criteria. **Phase 2:** Introduce financing and reimbursement mechanisms with financial monitoring. **Phase 3:** Evaluate costs, financial protection, equity, and sustainability and adjust financing mechanisms accordingly.	Budgetary constraints; competing healthcare priorities; political changes.	Annual public expenditure on ART; public expenditure per ART cycle; cost per live birth; proportion of total ART-related costs covered through public financing; median patient out-of-pocket expenditure per treatment cycle; incidence of catastrophic health expenditure related to infertility care; annual program budget execution rate.

ART, assisted reproductive technology; IVF, *in vitro* fertilization; ICSI, intracytoplasmic sperm injection.

## 6 Discussion

The incorporation of ART into publicly funded health systems represents one of the most important policy challenges in reproductive healthcare (1, 3, 4, 13, 18, 20, 23, 30, 31). While discussions have traditionally focused on financing mechanisms, legal regulation, and access to high-complexity reproductive procedures (2, 13, 23, 24, 30), comparatively less attention has been paid to the organization of service delivery across health system levels. This review argues that the effectiveness of public ART programs depends not only on expanding the availability of specialized procedures but also on developing integrated care models capable of ensuring equitable access, continuity of care, and efficient resource allocation (6, 8, 20–22, 31).

A central finding of this review is that infertility should be approached as a chronic care pathway rather than as an isolated specialized intervention. Individuals seeking fertility care often require multiple healthcare interactions before referral to specialized centers and continue to need coordinated care following ART treatment, particularly during pregnancy and the postpartum period (16, 17, 25, 26). Consequently, organizational models that rely exclusively on tertiary fertility centers may overlook substantial opportunities to improve care coordination and patient experience throughout the reproductive continuum (21, 25, 27, 28).

Integrating secondary-level hospitals into ART service delivery networks offers a pragmatic strategy for strengthening continuity of care while preserving the technical advantages of centralized high-complexity procedures (4, 21, 31, 34). Rather than decentralizing IVF or other advanced reproductive technologies, this model proposes an appropriate distribution of responsibilities according to institutional capacity. Secondary-level hospitals may contribute through infertility assessment, diagnostic evaluation, patient counseling, referral coordination, follow-up after treatment, and pregnancy surveillance, whereas tertiary fertility centers remain responsible for laboratory-intensive reproductive interventions (17, 21, 25, 32, 33, 36). Such functional integration may simultaneously improve accessibility, optimize specialized resources, and reduce unnecessary fragmentation within the healthcare system (6, 8, 22, 32, 33, 35).

Importantly, the potential benefits attributed to the proposed network-based model should be distinguished from effects that have been empirically demonstrated. The available literature supports the broader principles underlying integrated care, including coordinated referral pathways, continuity across levels of care, and the concentration of highly specialized procedures in appropriately equipped centers (50, 51). However, direct empirical evidence demonstrating that the specific integration of secondary-level hospitals into publicly funded ART networks improves equity, reduces waiting times or patient costs, increases referral completion, or improves reproductive outcomes remains limited (52).

From a health systems perspective, the proposed model also supports broader objectives of universal health coverage by promoting more coordinated, person-centered, and territorially accessible healthcare services (9–12, 20, 35). These organizational principles are consistent with contemporary approaches to integrated care, which emphasize collaboration between different levels of care, multidisciplinary management, and continuity across the patient journey (25, 32, 33, 35, 36). Although implementation strategies will necessarily vary according to national context (7, 18, 30, 34), strengthening coordination between secondary and tertiary care may represent a feasible organizational approach for countries seeking to expand publicly funded ART services while maintaining sustainability (1, 4, 18, 31, 34).

International experience demonstrates substantial variation in how ART is financed and delivered, ranging from predominantly publicly funded systems to models that rely largely on private provision and out-of-pocket payment (32). Several European countries provide broad public or social-insurance coverage, although treatment may be delivered through public, private, or mixed networks; France and Belgium, for example, reimburse eligible ART treatment even when services are provided by private centers. Argentina and Uruguay have similarly developed comparatively comprehensive public frameworks in Latin America, whereas Brazil and Chile provide publicly supported treatment with more limited availability. Importantly, public coverage does not necessarily guarantee effective access. In Brazil, limited numbers of public ART centers, geographic concentration, and long waiting lists demonstrate how insufficient service capacity can restrict access despite inclusion within the public health system (19, 53, 54).

In contrast, where ART remains predominantly privately financed, access is more directly determined by patients' ability to pay. This pattern remains relevant in several Latin American countries, including Ecuador, where ART provision is largely concentrated in the private sector and comprehensive public coverage remains limited (54). International comparisons have shown that greater out-of-pocket expenditure is associated with lower utilization of ART, illustrating the importance of financial protection in determining effective access (55).

Successful implementation, however, requires addressing several operational challenges. Clear governance structures, standardized referral and counter-referral pathways, interoperable health information systems, sustainable financing mechanisms, and appropriately trained healthcare professionals are essential to ensure that integrated service delivery translates into measurable improvements in access and quality (19, 23, 29–34, 36). Likewise, monitoring systems should extend beyond conventional clinical outcomes to include indicators of equity, continuity of care, patient experience, and health system performance, thereby providing policymakers with a more comprehensive assessment of program effectiveness (5, 10, 14, 15, 19, 20, 29, 35).

The recommendations presented in this review were developed through a policy-oriented interpretation of the evidence identified in the literature. The available evidence on access to infertility care, integrated service delivery, referral pathways, continuity of care, equity, governance, financing, and health information systems provided the basis for identifying the main organizational challenges and potential policy responses. This evidence was subsequently analyzed within the Public Policy Observatory of Hospital Provincial General Pablo Arturo Suárez, with contributions from public health experts affiliated with several universities in Ecuador. This expert input supported the contextual interpretation of the evidence from a health-system and public policy perspective and informed its translation into the actionable recommendations and implementation framework presented in Table 1. The process was intended to complement, rather than replace, the evidence identified in the literature and did not constitute a formal expert-consensus method.

### 6.1 Limitations

This review has several limitations. First, it presents a policy-oriented analysis rather than a systematic review of effectiveness studies, and therefore does not quantitatively compare alternative organizational models. Second, evidence regarding the organization of publicly funded ART services remains heterogeneous across countries because healthcare financing arrangements, regulatory frameworks, and health system structures differ substantially (4, 13, 18, 23, 30). Consequently, some recommendations may require adaptation to local institutional capacity and resource availability (1, 18, 30, 34). Third, patients and frontline healthcare staff were not directly consulted as part of this review; therefore, their perspectives were not empirically incorporated into the evaluation of the proposed organizational model or the development of the recommendations. Although the recommendations were informed by the available literature and further contextualized through contributions from public health experts, this process did not include direct patient, community, or frontline healthcare staff participation. This limitation is particularly relevant because these stakeholders may identify barriers related to accessibility, acceptability, care coordination, workload, feasibility, and continuity of care that are not fully captured in the published literature or expert policy analysis. Finally, empirical evidence evaluating the impact of integrated ART networks on equity, efficiency, and long-term reproductive outcomes remains limited, highlighting the need for implementation research and comparative policy evaluations (14, 20, 31).

Future research should therefore examine how different organizational models influence access to infertility care, patient outcomes, healthcare costs, and system performance across diverse settings (6, 8, 14, 22–24, 31). Comparative analyses of integrated reproductive care networks, economic evaluations, and implementation studies will be particularly valuable for informing evidence-based policy development (14, 18, 31, 34). Future implementation and evaluation studies should also directly incorporate patient, community, and frontline healthcare staff perspectives through stakeholder consultation and appropriate qualitative and quantitative methods. Such engagement would allow the feasibility, acceptability, accessibility, and responsiveness of the proposed organizational strategies to be assessed and could inform their adaptation to local contexts. In addition, the establishment of national ART registries may facilitate international benchmarking and contribute to generating robust evidence to guide future reforms in reproductive healthcare delivery (15, 16, 29).

Overall, expanding ART within the public sector should be understood as a health system reform rather than solely as the introduction of a specialized clinical service (1, 4, 18, 20, 30, 31). Policies that strengthen coordination between different levels of care, promote equitable access, and support continuous monitoring of clinical and organizational outcomes may contribute to more sustainable improvements in infertility care (5, 6, 19–22, 31). Integrating secondary-level hospitals into coordinated ART networks represents one promising policy option for advancing these objectives while reinforcing the principles of equity, continuity, and person-centered care that underpin modern public health systems (20, 21, 25, 31, 35).

The proposed model is directly applicable to the Ecuadorian context because it was conceptualized within the organizational principles of the country's Family, Community, and Intercultural Comprehensive Health Care Model (MAIS-FCI) and its differentiated levels of care (56, 57). Ecuador already distinguishes secondary- and tertiary-level hospitals according to their functions and complexity, providing an existing organizational structure through which responsibilities for infertility care could be distributed. Within this framework, secondary-level hospitals could undertake infertility assessment, diagnostic evaluation, patient navigation, referral and counter-referral coordination, follow-up, and pregnancy surveillance, while tertiary-level centers with specialized reproductive capacity would remain responsible for laboratory-intensive and high-complexity ART procedures. Therefore, implementation would build on the existing organization of the health system rather than require the creation of an entirely new level of care.

Although developed with the Ecuadorian health system in mind, the model may be transferable to other settings with differentiated levels of care or hospital categories, including basic, general, and specialized hospitals. Transferability depends on the capacity to distribute complementary functions according to institutional complexity, maintain effective referral and counter-referral pathways, and ensure access to specialized ART centers. Specific responsibilities, financing, workforce, and implementation mechanisms should therefore be adapted to each health system's organization and available resources.

## 7 Conclusion

Expanding ART within publicly funded health systems requires more than financing high-complexity reproductive procedures. It demands organizational models capable of ensuring equitable access, continuity of care, efficient resource allocation, and coordinated service delivery across the healthcare network. As countries continue to develop legislative and regulatory frameworks for infertility care, equal attention should be given to the design of health service delivery models that can translate policy commitments into accessible and sustainable clinical practice.

This review highlights the potential contribution of secondary-level hospitals as strategic components of integrated ART networks. By supporting infertility assessment, diagnostic evaluation, referral coordination, longitudinal follow-up, pregnancy surveillance, and collaboration with specialized fertility centers, these facilities may strengthen the continuum of reproductive care while allowing tertiary centers to focus on advanced ART procedures. Such an organizational approach has the potential to improve equity, optimize the use of specialized resources, and enhance the overall performance of public infertility programs.

Ultimately, the successful integration of ART into public health systems should be understood as a health system strengthening strategy rather than the expansion of a single specialized service. Future public policies should prioritize coordinated governance, standardized referral pathways, workforce development, robust health information systems, and continuous monitoring of clinical and equity outcomes. Adopting integrated network-based models may provide a practical and sustainable pathway toward universal, person-centered infertility care, ensuring that advances in reproductive medicine translate into meaningful improvements in access, quality, and reproductive equity.
